# The BALA project: A pioneering monitoring of Azorean forest invertebrates over two decades (1999–2022)

**DOI:** 10.1038/s41597-024-03174-7

**Published:** 2024-04-11

**Authors:** Gabor Pozsgai, Sébastien Lhoumeau, Isabel R. Amorim, Mário Boieiro, Pedro Cardoso, Ricardo Costa, Maria Teresa Ferreira, Abrão Leite, Jagoba Malumbres-Olarte, Guilherme Oyarzabal, François Rigal, Alejandra Ros-Prieto, Ana M. C. Santos, Rosalina Gabriel, Paulo A. V. Borges

**Affiliations:** 1 cE3c - Centre for Ecology, Evolution and Environmental Changes & CHANGE - Global Change and Sustainability Institute, Faculty of Agricultural Sciences and Environment, University of the Azores, Rua Capitão João d´Ávila, Pico da Urze, 9700-042 Angra do Heroísmo, Portugal; 2 IUCN SSC Atlantic Islands Invertebrates Specialist Group, 9700-042 Angra do Heroísmo, Azores Portugal; 3 cE3c- Centre for Ecology, Evolution and Environmental Changes, CHANGE – Global Change and Sustainability Institute, Faculty of Sciences, University of Lisbon, Lisbon, Portugal; 4 LIBRe – Laboratory for Integrative Biodiversity Research, Finnish Museum of Natural History, University of Helsinki, P.O.Box 17 (Pohjoinen Rautatiekatu 13), 00014 Helsinki, Finland; 5 Regional Secretariat of Environment and Climate Change, Project LIFE BEETLES (LIFE 18NAT/PT/000864), Rua do Galo n118, 9700-040 Angra do Heroísmo, Azores Portugal; 6 Rua Fernando Pessoa, n°99 R/C DTO 2765-483, Estoril, Portugal; 7 Institut Des Sciences Analytiques et de Physico Chimie pour L’environnement et les Materiaux UMR5254, Comité National de la Recherche Scientifique - University de Pau et des Pays de l’Adour - E2S UPPA, Pau, France; 8 Terrestrial Ecology Group (TEG-UAM), Departamento de Ecología, Universidad Autónoma de Madrid, 28049 Madrid, Spain; 9 Centro de Investigación en Biodiversidad y Cambio Global (CIBC-UAM), Universidad Autónoma de Madrid, 28049 Madrid, Spain; 10 IUCN SSC Species Monitoring Specialist Group, 9700-042 Angra do Heroísmo, Azores Portugal

**Keywords:** Biodiversity, Macroecology, Invasive species

## Abstract

Globally, there is a concerning decline in many insect populations, and this trend likely extends to all arthropods, potentially impacting unique island biota. Native non-endemic and endemic species on islands are under threat due to habitat destruction, with the introduction of exotic, and potentially invasive, species, further contributing to this decline. While long-term studies of plants and vertebrate fauna are available, long-term arthropod datasets are limited, hindering comparisons with better-studied taxa. The Biodiversity of Arthropods of the Laurisilva of the Azores (BALA) project has allowed gathering comprehensive data since 1997 in the Azorean Islands (Portugal), using standardised sampling methods across islands. The dataset includes arthropod counts from epigean (pitfall traps) and canopy-dwelling (beating samples) communities, enriched with species information, biogeographic origins, and IUCN categories. Metadata associated with the sample protocol and events, like sample identifier, archive number, sampled tree species, and trap type are also recorded. The database is available in multiple formats, including Darwin Core, which facilitates the ecological analysis of pressing environmental concerns, such as arthropod population declines and biological invasions.

## Background & Summary

Conserving the biodiversity of island biotas is a global concern because of the unique set of species living on islands^1^. Most native floras and faunas on islands face great challenges^2,3^, with intensifying land use changes which lead to the destruction of habitats, and the increasing number of introduced, and potentially invasive, species which act as predators, parasites, or competitors to the native ones^4,5^, being the most worrisome. In this context, highly specialised and small-range endemic species are particularly vulnerable^6^. Although the results obtained with long-term arthropod datasets can strongly support the understanding of the vulnerability of the endemic ecosystems, plant and vertebrate population trends, including extinctions, are still relatively better-documented^7,8^ than to those of arthropods. Yet, for instance, alien insect species were reported to threaten endemic diversity on the Galapagos Islands^9^ and negative correlation was found between invasive ants and the species richness of other insect taxa on island of the Great Barrier Reef^10^. Most of these studies, however, rely on short-term datasets or qualitative historical data.

Indeed, despite their key role in ecosystem functioning and the high conservation importance of endemic species, arthropod diversity in most archipelagos is substantially understudied, with studies spanning long periods being rare^11^.

**Fig. 1:**
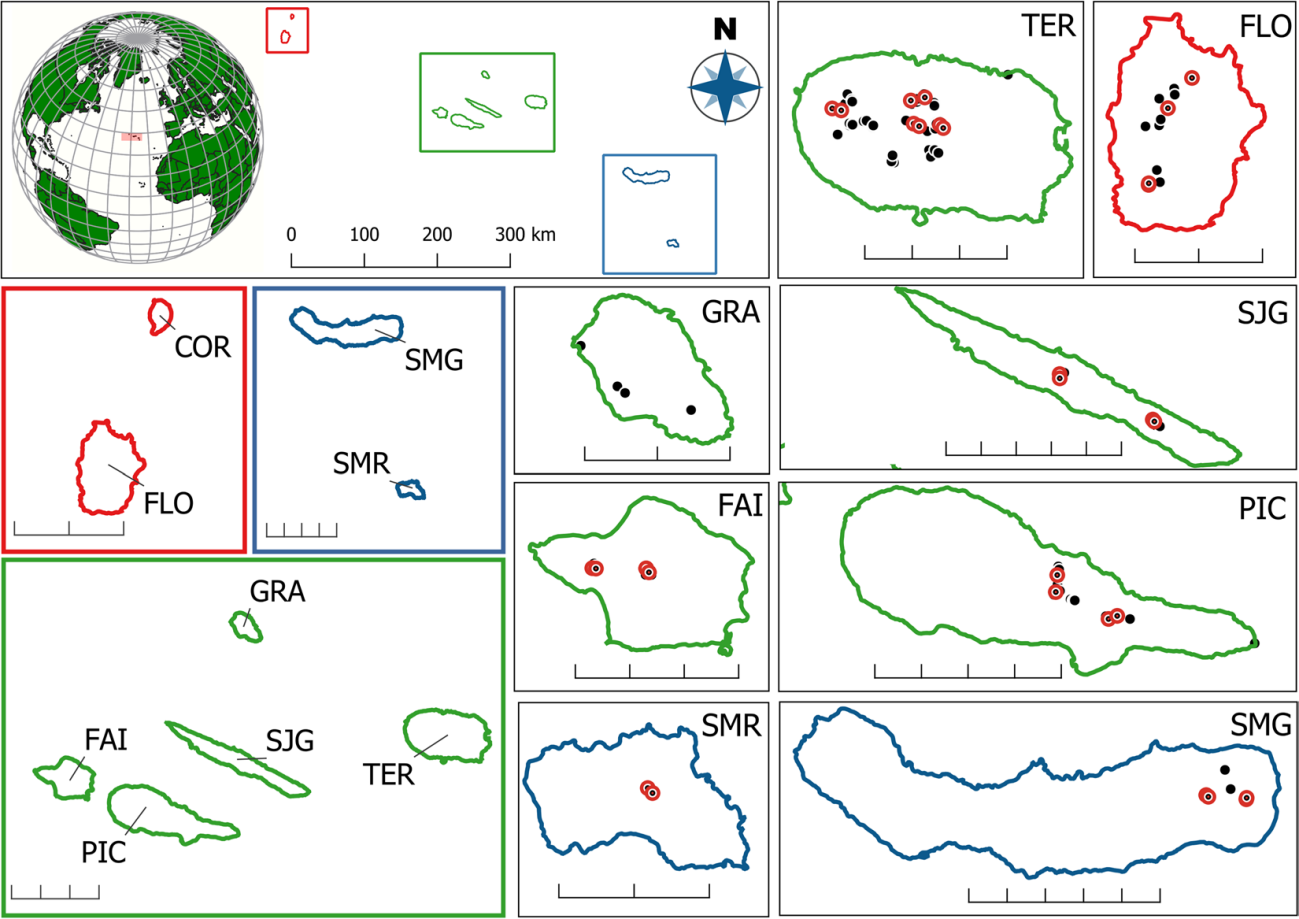
Map of the Azores archipelago with the location of sampling transects. Top left shows the position of the three island groups (Western group – red, Central group – green, and Eastern group – blue). The three groups are shown separately on the left side and the individual islands with all sampling points (black dots) and core sampling points (red circles) are shown on the right. Abbreviations as: FLO – Flores, COR – Corvo, FAI – Faial, PIC – Pico, SJG – São Jorge, GRA – Graciosa, TER – Terceira, SMG – São Miguel, and SMR – Santa Maria. Note that no pristine area remains in Graciosa and Corvo and there was no sampling on Corvo. Scales on the group maps show 20 km, and 10 km on the maps of individual islands.

The BALA ‘Biodiversity of Arthropods of the Laurisilva of the Azores’ dataset aims to address this gap in knowledge. It contains a unique and comprehensive set of long-term quantitative arthropod data, sampled over 3 sampling campaigns covering 25 years and across eight Azorean islands (Fig. 1), using consistent and standardised sampling protocols.

The core dataset is a result of 4,929 sampling events of 31 transects distributed across 15 fragments of native Azorean humid forests on 7 islands (excluding Corvo and Graciosa). Sampling was conducted between 1997 and 2022 and split into three multi-year sampling campaigns, BALA 1 (between 1997 and 2004), BALA 2 (2010 and 2011), and BALA 3 (between 2019 and 2022). The core dataset contains only transects in pristine native vegetation that have been repeatedly sampled during this period, but the final dataset also includes data from several other projects that used the same methodology to sample invertebrates across the archipelago. The final dataset thus encompasses data of 124 transects in 27 fragments from eight islands (Table 1). Most fragments are pristine native forests but, with the extended dataset, the early succession of lava flows and secondary forests (on Graciosa island, where no native habitat is left) are also included.

**Table 1:** Numbers of transects sampled per year in each fragment.

Island	Fragment name	1997	1999	2000	2001	2002	2003	2004	2005	2010	2011	2019	2020	2021	2022
		BALA 1								BALA 2		BALA 3			
**FAI**	Cabeço do Fogo		2					2		2				2	
	Caldeira do Faial							4		4				2	
**FLO**	Caldeiras Funda e Rasa		2	2						2			2		
	Morro Alto e Pico da Sé		4	4						2			2		
**GRA**	Barro Branco								1						
	Caldeira								1						
	Caldeiras								1						
	Caldeirinha								1						
	Quitadouro								1						
**PIC**	Manhenha		1												
	Caveiro		2	2						2			2		
	Lagoa do Caiado		2	2											
	Mistério da Prainha		4	4						2			2		
**SJG**	Pico Pinheiro		1	1				2		2					2
	Topo		1	1				2		2					2
**SMG**	Atalhada		2	1				2							
	Graminhais		2	1				2		2					2
	Pico da Vara		3	1						2					2
**SMR**	Pico Alto	2				2		2		2		2			2
**TER**	Algar do Carvão					2									
	Matela		2			2									
	Biscoito da Ferraria		2	1			8				2			2	
	Caldeira Guilherme Moniz					2	3								
	Pico Galhardo					2	3			2				2	
	Serra de Santa Bárbara		6		2		11			2			2		
	Terra Brava		2		2	2	7	1		2			1	1	

Island abbreviations: FAI = Faial, FLO = Flores, GRA = Graciosa, PIC = Pico, SJG = São Jorge, SMG = São Miguel, SMR = Santa Maria, TER = Terceira.

The core dataset includes samples collected in sub-montane and montane forests. The sub-montane forests are dominated by *Laurus azorica* (Azorean Laurel forests), that in the past probably covered more than two thirds of the islands, from 300 m to 600 a.s.l.^2^. The high elevation sites between 600 m and 1000 a.s.l. are Juniperus-Ilex forests and Juniperus woodlands^12^. All these forests are characterised by a dense cover of bryophytes that are present on all substrates.

**Fig. 2:**
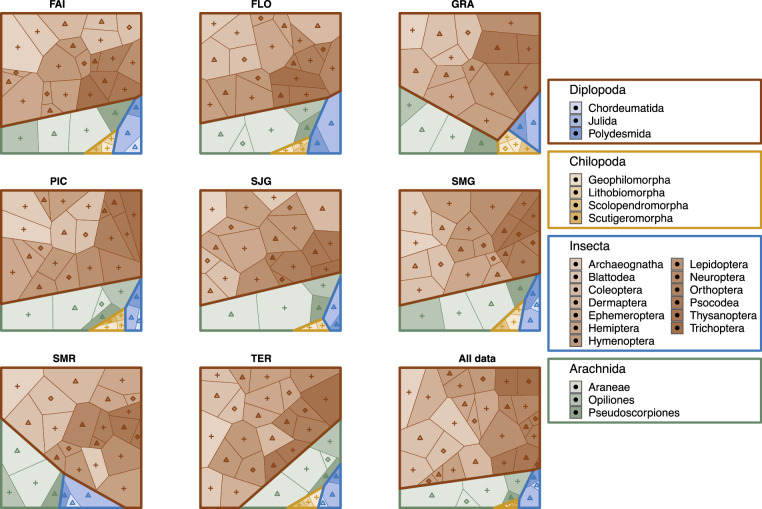
Voronoi maps showing the proportions of species richness of invertebrates caught in different higher taxa on each island (FAI = Faial, FLO = Flores, GRA = Graciosa, PIC = Pico, SJG = São Jorge, SMG = São Miguel, SMR = Santa Maria, TER = Terceira) and in the entire dataset (All data). Main colours represent arthropod classes, whilst different hues of these colours indicate families. Indigenous (endemic and native but not endemic), introduced species, and those of unknown origin are marked with patterns of triangles, squares, and crosses, respectively.

**Fig. 3:**
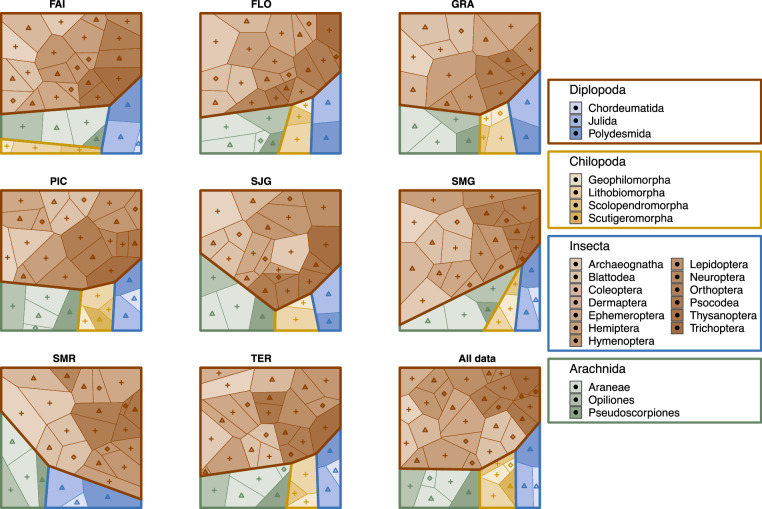
Voronoi maps showing the proportions of total log abundances of arthropods collected in different higher taxa on each island (FAI = Faial, FLO = Flores, GRA = Graciosa, PIC = Pico, SJG = São Jorge, SMG = São Miguel, SMR = Santa Maria, TER = Terceira) and in the entire dataset (All data). Main colours represent arthropod classes, whilst different hues of these colours indicate families. Indigenous (endemic and native but not endemic), introduced species, and those of unknown origin are marked with patterns of triangles, squares, and crosses, respectively.

Altogether, the collection amasses a total of 266,675 specimens belonging to 543 arthropod morphospecies (123,446 individuals and 404 morphospecies in the core dataset), of which 354 (65.2%) are identified at the species level. Of these species, 77 (105,919 individuals) are endemic to the Azores, 97 (97,562 individuals) are native but not endemic, and 146 (32,267 individuals) are exotics (Figs. 2, 3).

## Methods

### Study area and biogeographical aspects

The Azorean archipelago is situated in the North Atlantic Ocean, comprising nine volcanic islands and occupying a surface area of 2,346 km^2^ (Fig. 1). All islands have a wet oceanic, mild to warm, subtropical climate, with moderate variation in daily and annual temperatures^13^.

Biogeographically, the Azores belong to Macaronesia, with its native vegetation being characterised by several types of hyper-humid forests, including the laurel forests (Laurisilva)^12^. However, the islands have been inhabited since the mid-15^th^ century and the native forest, originally covering almost the entire surface of the islands, has been increasingly converted to agricultural cropland, pastures, settlements, and, during the 20^th^ century, tree plantations dominated by *Cryptomeria japonica* (Thunb. ex L.f.) D.Don. Even the remaining fragments are dominated by *Juniperus* forests and shrublands different from those of Madeira and Canary Islands^12^. By 1988, when Natural Forest Reserves were established on seven of the nine Azorean islands (see below), only 2.5% of the original native forests remained in isolated fragments^14–16^.

In our study, eight islands of the Azorean archipelago (from west to east) were sampled: Flores, Faial, Pico, São Jorge, Graciosa, Terceira, São Miguel, and Santa Maria. The surveyed native vegetation included (according to reference^12^): *Laurus* Submontane Forest*, Laurus*-*Ilex* forests (300–600 m), that hosted 14 woody plant species in average, with a tree height ranging from 6 to 8 m, being dominated by *Laurus azorica*; *Juniperus*-*Ilex* Montane Forests (600–900 m), that hosted an average of 18 plant species, with a tree height ranging from 3 to 5 m, being dominated by *Juniperus brevifolia* and *Ilex azorica* but *Laurus azorica* being still very frequent; and *Juniperus* Montane Woodlands between 700 m and 1000 m, hosting an average of 15 plant species, with a tree height ranging from 2 to 4 m, being dominated by *Juniperus brevifolia.* In the special case of the lower elevation island of Santa Maria, the native forest is a mix of *Picconia-Morella* lowland forest and *Laurus* Submontane Forest.

No pristine area remained in Graciosa and thus on this island only secondary forests could be sampled using the sampling protocol introduced below. Corvo island has no pristine vegetation either and its remoteness makes sampling logistically challenging, thus this was not sampled.

### Projects’ details

The dataset stems from a number of projects using the same sampling protocol for collecting arthropods in the Azorean archipelago. The Biodiversity of Arthropods from the Laurisilva of the Azores (BALA) project was established with a vision to initiate a comprehensive survey of the Azorean invertebrate fauna, with a particular interest in endemic species^17^. The main initial goals were to: (i) contribute to solve the Linnaean Shortfall^18^, by surveying new habitats (tree canopy) and formally describing new species (taxonomic effort); (ii) gather comprehensive and sustained ecological data and to meticulously assess the spatial and temporal dynamics of species distributions and abundance across various scales. The initiative also aims to (iii) address the gaps highlighted by the Wallacean and Prestonian shortfalls^19^; and, by providing a thorough understanding of ecosystem dynamics and facilitating informed conservation strategies, (iv) inform the Azorean Government about the quality status of the Azorean native forest.

Later, with the two consecutive sampling campaigns over the next 20 years, it became the longest running monitoring effort of the changes of the Azorean biota. Its relatively large scale both in space and time also made the BALA dataset suitable for testing macroecological and biogeographical hypotheses (e.g. refs. ^15,20^) and also contributing to clarify the potential occurrence of an “insect decline” in the Azores^5^ and identify the spatial and temporal invasion patterns of exotic arthropod species. The Project is coordinated by the Azorean Biodiversity Group (cE3c), based at the University of the Azores in Angra do Heroismo, Terceira.

The core sampling, organised into three consecutive phases, sampled the same 30 sites repeatedly. However, the core database includes 31 sites (Table 2) because one of the sites surveyed during BALA 1 in Faial island (FAI-NFCF-T-11) had to be replaced (FAI-NFCF-TB26) with a nearby location due to an invasion by *Rubus ulmifolius* Schott that made the installation of BALA 2 and BALA 3 pitfall traps impossible.

**Table 2:** The 31 core BALA sites. Island abbreviations: FAI = Faial, FLO = Flores, GRA = Graciosa, PIC = Pico, SJG = São Jorge, SMG = São Miguel, SMR = Santa Maria, TER = Terceira.

Site code	Island	Fragment_Name	Specific habitat type	Area	Perimeter	Altitude	Latitude	Longitude
FLO-NFFR-T-07	FLO	NFF Caldeiras Funda e Rasa	Native forest (C)	240	14658	528	39.40625	−31.22811
FLO-NFFR-T-06	FLO	NFF Caldeiras Funda e Rasa	Native forest (D)	240	14658	540	39.40736	−31.22800
FLO-NFMA-T-08	FLO	NFF Morro Alto e Pico da Sé	Native forest (D)	1331	29595	776	39.46070	−31.21377
FLO-NFMA-T-16	FLO	NFF Morro Alto e Pico da Sé	Native forest (D)	1331	29595	651	39.48269	−31.19314
FAI-NFCG-T-01	FAI	NFF Cabeço do Fogo	Erica on lava flow	36	2232	437	38.58506	−28.76803
FAI-NFCG-T-03	FAI	NFF Cabeço do Fogo	Native forest (A)	36	2232	512	38.58478	−28.76420
FAI-NFCF-TB26	FAI	NFF Caldeira do Faial	Native forest (C)			573	38.58567	−28.71121
FAI-NFCF-T-10	FAI	NFF Caldeira do Faial	Native forest (D)	191	11360	715	38.58251	−28.70823
PIC-NFMP-T-10	PIC	NFF Mistério da Prainha	Erica on lava flow	689	19735	771	38.46224	−28.27475
PIC-NFMP-T-01	PIC	NFF Mistério da Prainha	Native forest (B)	689	19735	656	38.47863	−28.27327
PIC-NFCA-T-09	PIC	NFF Caveiro	Native forest (D)	184	11128	940	38.43701	−28.20948
PIC-NFCA-T-08	PIC	NFF Caveiro	Native forest (D)	184	11128	896	38.44008	−28.19884
SJG-NFPP-T-02	SJG	NFF Pico Pinheiro	Native forest (C)	74	5568	665	38.64953	−28.04935
SJG-NFPP-T-09	SJG	NFF Pico Pinheiro	Erica on semi-natural pasture	74	5568	725	38.64409	−28.04909
SJG-NFTO-T-12	SJG	NFF Topo	Native forest (D)	220	10692	824	38.59258	−27.89614
SJG-NFTO-T-06	SJG	NFF Topo	Native forest (D)	220	10692	825	38.59012	−27.89345
TER-NFSB-T-06	TER	NFF Serra de Santa Bárbara	Native forest (D)	1347	25296	786	38.74981	−27.33169
TER-NFSB-T-11	TER	NFF Serra de Santa Bárbara	Native forest (C)	1347	25296	912	38.74803	−27.32074
TER-NFBF-T-02	TER	NFF Biscoito da Ferraria	Native forest (C)	557	18060	575	38.75757	−27.23628
TER-NFPG-T-22	TER	NFF Pico Galhardo	Native forest (B)	38	5012	589	38.73567	−27.23283
TER-NFPG-T-33	TER	NFF Pico Galhardo	Native forest (B)	38	5012	651	38.73329	−27.22609
TER-NFBF-T-01	TER	NFF Biscoito da Ferraria	Native forest (D)	557	18060	694	38.76055	−27.21927
TER-NFTB-T-15	TER	NFF Terra Brava	Native forest (B)	180	8480	639	38.73445	−27.20091
TER-NFTB-T-18	TER	NFF Terra Brava	Native forest (B)	180	8480	668	38.73159	−27.19683
SMG-NFGR-T-03	SMG	NFF Graminhais	Native forest (D)	16	2366	910	37.80174	−25.24329
SMG-NFGR-T-07	SMG	NFF Graminhais	Native forest (D)	16	2366	932	37.79974	−25.24014
SMG-NFPV-T-01	SMG	NFF Pico da Vara	Native forest (C)	306	9555	646	37.79598	−25.18381
SMG-NFPV-T-04	SMG	NFF Pico da Vara	Native forest (C)	306	9555	586	37.79761	−25.18327
SMR-NFPA-T-01	SMR	NFF Pico Alto	Native forest (A; B)	9	2203	504	36.97949	−25.08970
SMR-NFPA-T-03	SMR	NFF Pico Alto	Native forest (A; B)	9	2203	429	36.97636	−25.08607

A - *Picconia*-*Morella* lowland (100–300 m); B - *Laurus* Sub-montane Forest, *Laurus*-*Ilex* forests (300–600 m); C - *Juniperus*-*Ilex* Montane Forests (600–900 m); D - *Juniperus* Montane Woodlands between 700 m and 1000 m.

However, in the initial sampling round, which took place from 1997 to 2004 (referred to as BALA 1), a total of 100 sites within 18 forest fragments were surveyed as a result of a collective effort from several projects. During the subsequent rounds, spanning from 2010 to 2012 (BALA 2^21^) and 2021 to 2022 (BALA 3), only the 30 core sites, out of the original 100 sites, located within 15 fragments were resampled. Although no pristine native vegetation is found on the small island of Graciosa, a single expedition was conducted in June 2005 to survey 11 sites on this island (Project BALA Graciosa). The Geotermia1 project, also using the BALA protocol, was a monitoring survey of native forest plots in Terceira Island, aiming to evaluate the impact of the construction of a geothermal power plant near Galhardo Natural Forest Fragment. These surveys were conducted in 2002 and 2007 in 16 native forest sites. Data from two PhD projects are also included in the dataset. In the first, by Clara Gaspar (2003-2004), 44 additional BALA 1 plots were setup and in the second, by Silvia Calvo Aranda, this sampling at the same sites was repeated during BALA 2 (2011) (Table 1).

### Sampling protocol

Each site was sampled for soil fauna along a 150 m transect, in which 30 pitfall traps, each with a 5 cm opening diameter, were placed at 5-meter intervals. Every second trap was filled with ethylene-glycol and the remaining 15 traps with Turquin’s solution (10 g chloral hydrate, 5 ml formalin, 5 ml acetic acid, added to 1 L of dark beer)^22^. Pitfall traps were collected after two weeks (14 nights) of continuous operation. Additionally, for the purpose of capturing arthropods residing in the canopy, the study also included ten samples per each of the three most common native tree species using a beating technique, primarily focusing on endemic *Juniperus brevifolia* (Seub.) Antoine (Cupressaceae), *Erica azorica* Hochst. ex Seub. (Ericaceae), *Ilex azorica* Gand. (Aquifoliaceae), *Laurus azorica* (Seub.) Franco (Lauraceae), and *Vaccinium cylindraceum* Sm. (Ericaceae). Trees were selected randomly within a 5 m distance from the pitfall trap line and were beaten five times at the height of ca. 1.5–2 m. Sampling campaigns took place between July-September when arthropods are the most active and canopy samples were always collected in dry and warm weather conditions. For examples when the BALA protocol was used, readers should consult the works using the BALA protocoll^14,17,23^.

### Taxonomic scope, biogeographic origin and conservation categorization

All arthropod taxa, with the exclusion of mites (Acari) and Collembola, were collected. Specimens were categorised to morphospecies on a first step and later identified to the lowest taxonomic level possible. Due to the difficulty of their identification, Diptera and Hymenoptera (excluding Formicidae) were not sorted to morphospecies but kept as bulk samples. Species identification was based on either the consultation of historical Azorean entomological literature, mostly for the identification of endemic species (see list of references in Borges & Vieira^24^) or the expertise of several taxonomists that collaborated with us in publications during the last 20 years (see e.g. ref. ^17^). Species nomenclature follows the last checklist of Azorean Arthropods^25^ and, with the exception of a few cases, it aligns with the GBIF Taxonomic Backbone. All identified species were categorised according to their biogeographic origin as 1) endemics to the Azores; 2) native but not endemic species, which occur naturally in the islands; and 3) introduced species whose presence is thought to be the result of (intentional or unintentional) human introduction^14,26^. The conservation status of each endemic species was assessed following the guidelines of the International Union for Conservation of Nature (IUCN) and an IUCN category was assigned to each.

### Sample and voucher archives

Upon collection, samples were labelled and initially stored in 96% ethanol. Identified specimens were either stored in 96% ethanol or mounted on insect pins. All samples were archived and voucher specimens preserved in the Dalberto Teixeira Pombo Insect Collection (Collection Code: DTP; collectionID: 1366b359-8936-4e40-be36-1f1e1eb6d2b0), situated at the University of the Azores in Angra do Heroismo, Terceira Island, Portugal. Pin-mounted specimens are kept at room temperature, with controlled humidity and safe from museum pests in well-sealed insect boxes. Specimens preserved in alcohol are kept in fridges at 4°Cor in a temperature-controlled room at 13°C.

### Data processing

Unique identifiers were assigned to each voucher morphospecies and event IDs, used by the GBIF, were assigned as identifiers to samples.

Morphospecies identifiers act as references and ensure that both voucher specimens can be traced back to their source and new identifications can be added or existing ones updated. By using the event ID, all metadata about the sampling event, such as when and where the sample was taken, by whom, can be retrieved. This also facilitates any corrections or updates to the database, as changes can be linked directly to specific events through their event IDs. Morphospecies occurrences and the abundances of juveniles, adults, males and females (when these were possible to identify) were linked to each event ID.

The dataset was cleaned up and converted to all formats using the *dplyr*^27^, *reshape2*^28^ and RMySQL^29^ and VoronoiPlus^30^ packages in R^31^. To make the Voronoi maps accessible to those with colour vision deficiency, their colours were chosen with the help of *colorblindr*^32^. All related code is deployed with the dataset and also available on the https://github.com/pozsgaig/BALA_database GitHub pages.

## Data Records

### Available formats

All data are available on figshare^33^ and also published on the GBIF website^34^ with the data fully compliant with the Darwin Core (DwC) standard (https://www.tdwg.org/standards/dwc), under a CC-BY 4.0 Licence.

The DwC is a standardised format developed to facilitate the sharing and integration of biological and biodiversity data across different platforms and databases, and provides a stable framework comprised of terms and vocabulary. This framework ensures that data from different sources remains interoperable and easily comprehensible, irrespective of its origin. The DwC may also encompass data related to sampling time, habitats, sampling methods, and other facets of biodiversity research.

To facilitate the usage of the data for those not familiar with the DwC format, we also provide one large data table, containing all data as semicolon separated values. Similarly, for those who wish to import data into GIS applications, we provide a geographically referenced relational database in a MySQL format.

### Database structure

The data consist of three separate data types: 1) data related with the sampling event, such as the sampling transect and its basic characteristics, date, and the sampling method; 2) morphospecies-related data, such as the species identity (if known), and higher taxonomy (i.e. genus, family, order, class), the biogeographic status, as well as the IUCN category; and 3) an occurrence dataset, including the number of individuals captured, separated to adults and juveniles and, when possible, to males and females.

DwC-formatted data consist of two tables. Whilst the event table contains information on the sampling event, such as sampling method, date, and site information, the occurrence table is focused on the organisms collected and it lists their taxonomic identity, biogeographic origin, and the abundance of the arthropods collected per development status (adults, juveniles) and sex, if it was determinable. The two tables are linked through the ‘eventID’ field unique to the event table. These two tables can be assessed at http://ipt.gbif.pt/ipt/resource?r = bala_arthropods^34^.

The MySQL database contains three separate tables: (1) the species list enriched with taxonomic information, biogeographic origin, and conservation status; (2) a sample site list, containing the WGS84 coordinates of the sampling sites, both as text and as a geometry field in MySQL; and (3) the occurrence table listing all collected specimens, method used for collection, life stage, and (where possible) sex. The species and site tables are linked to the occurrence table by the morphospecies ID and site ID, respectively. The database is provided in an.sql format which can directly be imported in any MySQL database. The tables are linked through unique identifiers, such as the morphospecies code and site code.

## Technical Validation

All samples were individually labelled and stored. Archive samples are still available for further data checks and analyses. Most species identifications were conducted by one of the authors (PAV Borges) but to some problematic specimens species-level identifications were assigned by expert taxonomists of the corresponding taxon.

Data were meticulously checked to avoid transcription errors and several tests were run to identify outliers in the data. For instance, unique values of each categorical variables were listed and carefully checked for misspellings, duplicates or similar errors and numerical variables and dates were plotted, and their interquartile range was examined and tested for outlying values. When necessary, these values were corrected or removed. Most of the data were already used in a number of analyses, for instance refs. ^21,35–37^.

All species names were automatically checked against the GBIF Taxonomy Backbone using the R function provided by Pozsgai *et al*.^38^.

## Usage Notes

Our database has great potential for analysing macroecological, biogeographical as well as species- and community-level patterns, particularly those focusing on insular systems. It can be especially valuable when combined with other island datasets. Indeed, since there are several long-term invertebrate datasets from Europe and the U.S.A.^39^, only a handful of those are available on island biotas (e.g. ref. ^40^). In addition to its primary objective of inventorying Azorean arthropods, the BALA database also allows comparison of diversities on multiple scales, at variable taxonomic level, and among a variety species groups (e.g. exotic and endemic species). Moreover, due to the long temporal span of the dataset, it also allows to contribute to biodiversity studies over time, namely investigating invasion dynamics and the effects of climate change and, hence, can support conservation planning.

## Data Availability

In order to eliminate possible mistakes, in the data format or any data fields, data were converted to all three data formats using a traceable computer code, which is openly accessible through the https://github.com/pozsgaig/BALA_database GitHub repository.
